# Bupleuri radix for Acute Uncomplicated Respiratory Tract Infection: A Systematic Review of Randomized Controlled Trials

**DOI:** 10.3389/fphar.2021.787084

**Published:** 2022-02-04

**Authors:** Li-Jiao Yan, Zhi-Jie Wang, Min Fang, Hui-Di Lan, Michael Moore, Merlin Willcox, Jeanne Trill, Xiao-Yang Hu, Jian-Ping Liu

**Affiliations:** ^1^ Centre for Evidence-Based Chinese Medicine, Beijing University of Chinese Medicine, Beijing, China; ^2^ Department of Oncology, Shanxi Provincial Hospital of Traditional Chinese Medicine, Shanxi, China; ^3^ Ruikang Hospital Affiliated to Guangxi University of Chinese Medicine, Nanning, China; ^4^ Primary Care and Population Sciences, Faculty of Medicine, University of Southampton, Southampton, United Kingdom; ^5^ Research Design Service South Central, National Institute of Health Research, Southampton, United Kingdom

**Keywords:** bupleuri radix, herbal medicine, Chinese herbal medicine, acute respiratory tract infection, systematic- review

## Abstract

**Objective:** To evaluate the efficacy, clinical effectiveness, and safety of the Chinese herb *Bupleuri radix* for the treatment of acute uncomplicated respiratory tract infections (ARTIs).

**Methods:** Four English and four Chinese databases were searched from their inception to June 2021. Randomized controlled trials (RCTs) assessing therapeutic effects of *Bupleuri radix* on ARTI were eligible for inclusion. The risk of bias for each trial was assessed using the Cochrane Risk of Bias Tool 2.0. RevMan 5.4 software was used for data analyses with effects estimated as risk ratios (RR) or mean differences (MD) with 95% confidence intervals (CI). The certainty of the evidence was assessed using the online GRADEpro tool.

**Results:** Seven randomized trials involving 910 patients with acute upper respiratory tract infection (AURTI) were included. The review identified *Bupleuri radix* agents with four administration routes (oral, acupoint injection, intramuscular injection, nebulized inhalation). *Bupleuri radix* acupoint injection compared with placebo showed statistically significant effects in reducing fever resolution time (MD: −33.32 h, 95%CI: −35.71, −30.93), and in increasing the proportion of participants with fever resolved within 48 h from treatment onset (RR: 14, 95%CI: 1.96, 99.94). *Bupleuri radix* acupoint injection combined with usual care is more effective in reducing the temperature at day 1 from treatment onset (MD: −1.00°C, 95%CI: −1.19, −0.81) compared with usual care alone. *Bupleuri radix* pills showed similar antipyretic effects to acetaminophen. However, *Bupleuri radix* intramuscular injection plus vitamins failed to demonstrate an effect in reducing fever, when compared with ribavirin plus vitamins. It suggested that oral administration of *Bupleuri radix* solution for injections, pills, and *Bupleuri radix* decoction have a similar effect on improving global AURTI symptoms including two key symptoms (nasal discharge and cough), when compared with usual care alone. Only two trials reported whether or not there were any AEs and found no occurrence of adverse events in the herbal group.

**Conclusion:** Low-certainty or very low-certainty evidence demonstrated that *Bupleuri radi*x (solution for injections and pills) has an antipyretic effect on febrile patients with AURTI, but it has no effect on other AURTI symptoms. However, these findings need to be further confirmed by well-designed clinical trials with adequate sample sizes.

**Systematic review registration:** (https://www.crd.york.ac.uk/prospero/#recordDetails), PROSPERO registration number: CRD42021234066.

## Introduction

Acute uncomplicated respiratory tract infections (ARTIs) involve both upper and lower airways; they include the common cold, influenza, otitis media, sinusitis, tonsillitis, laryngitis, pharyngitis, and bronchitis (Acute, 1998). Symptoms include nasal congestion and discharge, sneezing, sore throat, cough, sputum production, shortness of breath, chest pain, earache, and fever (Acute, 1998). Generally, typical common colds are self-limiting and last 7–10 days, whereas acute (rhino)sinusitis can last for up to 4 weeks (Acute, 1998). On average, acute bronchitis takes 3 weeks to resolve (NICE, N.I.f.H.a.C.E, 2010). ARTI is one of the most common reasons for primary care consultations. Treatments for ARTI are mainly symptomatic, and often include antipyretics, mucolytics, expectorants, decongestants, and educational interventions. Although ARTIs are predominantly viral infections and antibiotics show little benefit in symptom improvement for ARTI, antibiotics are frequently prescribed in primary care settings (Pouwels et al., 2018). Antimicrobial resistance (AMR) is an evolving major global threat to public health (Limmathurotsakul et al., 2019). The marginal benefit of antibiotics for ARTI is outweighed by increasing AMR and common adverse reactions leading to unnecessary increases in healthcare costs (Gonzales et al., 2001; Naylor et al., 2019). However, many patients believe in antibiotics and want a prescription (Cabral et al., 2015). Findings also suggest that many patients and doctors are willing to consider alternatives (Soilemezi et al., 2020; Willcox et al., 2020). Research is warranted to explore other alternatives that may offer symptomatic relief and reduce unnecessary antibiotic prescribing for ARTI.

The Chinese herb Chai hu (*Bupleuri radix*) is derived from the dried roots of *Bupleurum* L. There are approximately 200 genera and 2,500 species in different regions and herbal markets (Huang et al., 2017). Most notably the roots of *Bupleurum chinense* DC, *Bupleurum scorzonerifolium* Willd (China), and *Bupleurum falcatum* L (Japan) are commonly used in Traditional Chinese Medicine (TCM) and Kampo medicine.

With a 2000-years medicinal history, *Bupleuri radix* is believed to be one of the most important herbal medicines in China. The earliest record of *Bupleuri radix* in China appeared in the Divine Farmer’s Classic of Materia Medica (Han dynasty, 202 BC ∼ 220 AD) (Chen and Chen, 2004). Since then, *Bupleuri radix,* as an ingredient alone (Fang and Zhang, 2003) and within particular formulations (such as Chaihuang granule (Zhu et al., 2017)), has been widely used for the treatment of ARTIs in China, Japan, Korea, as well as other countries (Wyk and Wink, 2004; Klein et al., 2012; Yang et al., 2017). *Bupleuri radix* is often processed into pieces for easy use, but since the 1940s, *Bupleuri radix* injection has been formulated using steam distillation of the volatile oils from the herb. This is for treating influenza, the common cold, and malaria. With more recent developments of TCM, other *Bupleuri radix* preparations have been developed, such as pills and nasal sprays.

There is encouraging evidence demonstrating the potential mechanism for the effects of *Bupleuri radix* for ARTI. The active constituents of *Bupleuri radix* comprise mainly triterpenoid saponins, flavonoids, and essential oil (Yang et al., 2017). They possess anti-inflammatory activity by inhibiting some inflammation-associated cytokines, proteins, and enzymes, and regulating inflammation-related signal pathways. For example, the crude polysaccharides (80 mg/kg) isolated from the roots of *Bupleurum chinense* DC. significantly attenuated lung injury by inhibiting the activity of myeloperoxidase (MPO), reducing the production of tumor necrosis factor-α (TNF-α) in the bronchoalveolar lavage fluid (BALF) and of NO in serum (Xie et al., 2012). *Bupleuri radix* demonstrates antipyretic effects via adjustment of intracellular level of cyclic adenosine monophosphate (cAMP) and synthesis and exudation of arginine vasopressin (AVP). *Bupleuri radix* injection (5 ml/kg, 2.5 ml/kg, and 1.25 ml/kg) can significantly reduce the body temperature of rats (in the lipopolysaccharide fever model), and the dose-effect relationship is significant (Gao et al., 2012). It has also been shown to be effective against human coronavirus and influenza A virus through interference in the early stage of viral replication, such as absorption and penetration, and attenuating aberrant pro-inflammatory cytokine production *in vitro* (Yang et al., 2017). The ethanol extract of *Bupleurum chinense* DC. exerted a remarkable bacteriostatic effect on the Gram-negative microorganism *Helicobacter pylori in vitro* (Gao et al., 2012). The bioactive minimum inhibitory concentration (MIC) value was 60 μg/ml (Li et al., 2005). However, the toxic effects of *Bupleuri radix* in clinical applications have been gradually reported, especially for the preparation of *Bupleuri radix* injection. It has been implicated in multiple cases of acute hepatitis both as an ingredient alone and within a particular formulation “Xiao-Chai-Hu-Tang” (also known as Syo-Saiko-To in Japanese) (Itoh et al., 1995). A systematic review conducted in 2010 identified 203 ADR/AE cases in patients using *Radix Bupleuri* injection, such as anaphylactic shock, acute hepatitis, and acute hepatic necrosis, and for most intramuscular cases, ADR/AE happened within 30 min from injection (Kong et al., 2010). However, there is uncertainty about the side effects of other preparations of *Bupleuri radix*. Moreover, the Ministry of Health revised the Standards of *Bupleuri radix* injection (Administration, S.F.a.D., 2012) in 2011, which require it not to exceed 60 μg furfural (one of the main harmful ingredients) per 1 ml of the product. So far, there is uncertainty about the safety of *Bupleuri Radix* injection which follows the new standards.

## Objectives

This systematic review aims to evaluate the efficacy, clinical effectiveness, and safety of *Bupleuri radix* for the treatment of ARTI in randomized controlled trials (RCTs).

## Methods

### Criteria for Considering Studies for This Review

#### Types of Studies

All RCTs were eligible for inclusion.

#### Types of Participants

Trials with patients of any age, with either an ARTI diagnosis or symptoms of ARTI, were included. Diagnoses of ARTI included the common cold, influenza, rhinosinusitis, laryngitis, tonsillitis, pharyngitis, croup, acute otitis media, bronchitis, and acute exacerbations of chronic obstructive pulmonary disease (AECOPD). Symptoms of ARTI were defined as having symptoms such as cough, sore throat, fever, runny nose, and discolored sputum for less than 4 weeks (King et al., 2015).

We excluded any condition for which a specific therapy was recommended, such as streptococcal infections, pneumonia, diphtheria, tuberculosis, infections in immunocompromised, or any life-threatening condition. Also, studies restricted to patients with underlying chronic disease, such as asthma, or any other condition potentially impacting on the management and outcome of ARTI were not included.

#### Types of Interventions

Any form of preparation of *Bupleuri radix*, as monotherapy was included. Trials were included irrespective of the route of administration, e.g. oral, intramuscular injection or acupoint injection, or topical use. A preparation prescribed alone or as an adjunct treatment was only relevant if *Bupleuri radix* could be isolated as the intervention. Other treatments were permitted, such as additional symptomatic treatment, but this needed to follow national guidelines, and needed to be the same in both intervention and control groups. *Bupleuri radix* combined with other TCM therapies such as acupuncture were excluded.

#### Types of Control

No intervention, placebo; usual care such as antipyretics, antivirals, antibiotics, anti-inflammatories, steroids, or corticosteroids were included.

#### Prespecified Outcomes Included

Primary outcomes:
  1) Change in global symptoms, which is measured as time to complete resolution of global symptoms (in days) or the proportion of patients resolved at a predefined time.
  2) Change in some key symptoms (e.g. fever, cough, and sore throat), which measured as time to complete resolution of symptoms (in days), or the proportion of patients with symptoms resolved at a predefined time.
Secondary outcomes:
  1) Need for antibiotics at follow-up.
  2) Days off work or school.
  3) Length of hospitalization.
  4) Adverse events (AEs): These included any anaphylactic, allergic reactions, hypersensitivity reactions, or complications of taking *Bupleuri radix*. Information regarding AEs due to interactions of *Bupleuri radix* either as a monotherapy or in combination with other remedies, as well as potential interactions with medications for patients with comorbidities was collected.

We defined serious AEs according to the International Council on Harmonization of Technical Requirements for Registration of Pharmaceuticals for Human Use (ICH) guidelines as any event that leads to death, is life-threatening, requires hospitalization, or leads to persistent or significant disability, or leads to abnormal laboratory results such as liver or renal function tests (ICH, T.I.C.f.H., 1994).

### Search Methods for Identification of Studies

A wide range of sources were searched (by LJY and MF) to find both published and unpublished studies via the following electronic databases and grey literature sources from their inception to June 2021. The major Chinese electronic databases included China national knowledge infrastructure (CNKI), Chinese Scientific Journal Database (VIP), Chinese BioMedical Literature Database (Sinomed), and Wanfang Database (Wanfang). The international databases searched were: PubMed, the Cochrane Library (Issue 6), Embase, Allied, and Complementary Medicine Database, Web of Science, and trial registries via ClinicalTrials. The references of all identified reviews or clinical trials were searched for additional studies.

Search terms included “*Bupleuri radix*” AND “respiratory tract infection” AND “randomized controlled trials”. Additional search terms and strategies in different languages with different databases are listed in the Supplementary Material search strategy.

No language restrictions were applied.

### Study Selection and Data Extraction

All titles and abstracts of studies retrieved from the electronic searches were reviewed by two authors (LJY and MF), who selected the relevant articles by title and abstract. Full-texts of each publication were independently reviewed by the two authors to determine their inclusion based on the criteria. Two authors (LJY and HDL) independently carried out data extraction using a pre-tested data extraction form. A third author (JPL) resolved disagreements between the two authors in consultation with them. For included trials, we abstracted the following data as recommended in the Cochrane Handbook for Systematic Reviews of Interventions: 1) General information: published or unpublished, author, country, publication language, publication year, journal citation; 2) Participants: inclusion and exclusion criteria, the total number enrolled and number in each comparison group, baseline characteristics, setting; 3) Interventions: details of interventions in all trial arms including type and dose of therapy, according to the CONSORT 2010 extension for reporting Chinese herbal medicine formulas (CHM) checklist (Cheng et al., 2017); 4) Risk of bias in trials (see Assessment of risk of bias in included studies); 5) Follow-up: length of follow-up, the reason for and the number of dropouts and withdrawals, method of analysis; 6) Outcome measures, as the mean and standard deviation (SD) for continuous outcomes, and the number of events for dichotomous outcomes; 7) Safety and adverse events.

### Assessment of Risk of Bias in Included Studies

The risk of bias for each trial was assessed by using version 2 of the Cochrane tool for assessing the risk of bias in randomized trials (RoB 2) (Sterne et al., 2019). It included assessment of the randomization process, deviations from intended interventions, missing outcome data, measurement of the outcome, and selection of the reported result. Any disagreements were resolved by discussion.

### Measures of Treatment Effect

Statistical analyses were performed by using RevMan 5.4 (The Cochrane Collaboration, 2020). Dichotomous outcomes were expressed as risk ratio (RR) with 95% confidence interval (CI); Continuous data were presented as mean difference (MD) with 95% CI, or as standardized mean differences (SMDs) if outcomes were conceptually the same but measured in different ways in the different trials.

### Unit of Analysis Issues

The individual participant was the unit of analysis. For multiple treatment groups, we separated the arms into different comparisons that met our inclusion criteria. For example, one trial (Song, 2020) was multi-armed comparing *Bupleuri radix* injection with different administration routes (acupoint injection and intramuscular injection) versus placebo (acupoint injection with saline solution), and usual care (intramuscular injection with ribavirin). They were separated into two comparison groups: acupoint injection with *Bupleuri radix* versus acupoint injection with saline solution; and intramuscular injection with *Bupleuri radix* versus intramuscular injection with ribavirin.

### Dealing With Missing Data

We contacted authors where data was missing or incomplete. Where standard deviation was not reported with means, it was calculated from the information reported such as confidence intervals (CI), *p*-values, or F-values. The number of participants whose data was available at baseline and the last follow-up and the rate of loss to follow-up were recorded.

### Assessment of Heterogeneity

We planned to assess between-study heterogeneity using the I^2^ statistic which describes the percentage of variation across studies that is due to heterogeneity rather than chance. A rule of thumb for interpretation of this statistic suggests that I^2^ > 30% represents moderate heterogeneity, I^2^ > 50% represents substantial heterogeneity, and I^2^ > 75% represents considerable heterogeneity (Higgins et al., 2020). As high levels of heterogeneity were expected due to complexity in the form of *Bupleurum* (e.g. the various forms of preparation), a random-effects model was utilized to pool the overall effects.

### Assessment of Reporting Biases

We conducted funnel plot tests for asymmetry to investigate potential reporting bias where this was feasible and there were sufficient studies under a single meta-analysis.

### Data Synthesis

Where possible, the analyses were planned to be based on intention to treat (ITT) data on each outcome provided for every randomized participant from the individual trials. Where possible, for continuous outcomes, the end of treatment scores rather than change from baseline scores was extracted. Due to the expected variability in the populations and interventions of included trials, a generic inverse variance random-effects model was used to pool the data to incorporate heterogeneity.

### Subgroup Analysis and Investigation of Heterogeneity

We planned to conduct the following subgroup analyses for the primary outcome if there were sufficient trials:

• Adults (over 18) versus children
• *Bupleuri radix* in different preparations, e.g. granule versus capsule
• ARTI types regarding pathogen (bacterial or viral infection)

### Sensitivity Analysis

We planned to perform sensitivity analyses for the primary outcome to determine whether the conclusions were different if eligibility was restricted to trials with a low risk of overall bias.

Where substantial heterogeneity exists, sensitivity analysis was planned to be conducted to further investigate potential sources of heterogeneity.

## Results

### Results of the Search

**FIGURE 1:**
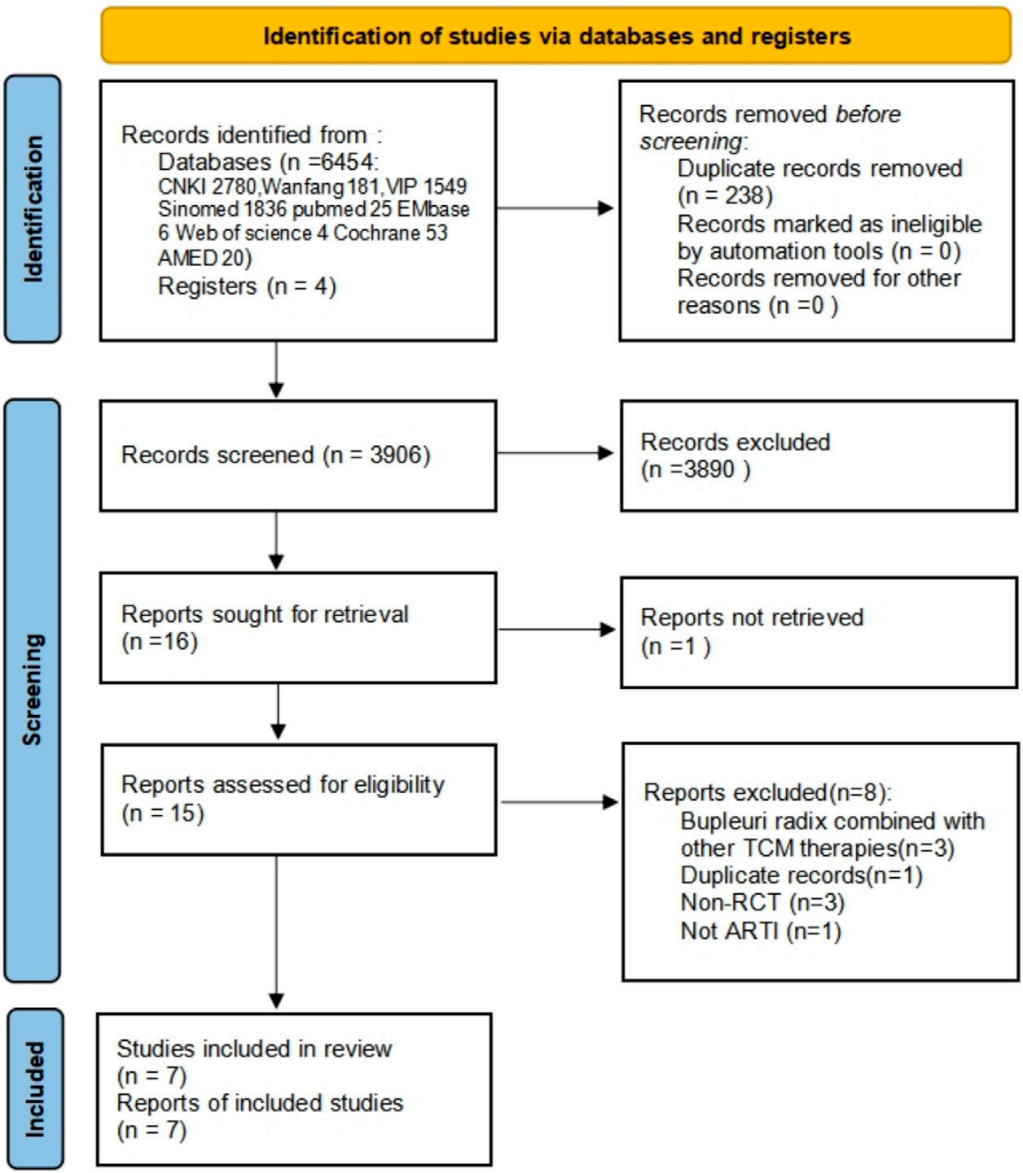
Flow and identification of trials included. CNKI: China national knowledge infrastructure; VIP: Chinese Scientific Journal Database; AMED: The Allied and Complementary Medicine Database; CBM: Chinese Bio Medical Literature Database; ARTI: Acute uncomplicated respiratory tract infections; RCT: randomized controlled trial.

In total 2,546 papers were identified, of which a total of 7 RCTs (Xu and Mao, 2001; Lv, 2010; Li et al., 2011; Cao, 2012; Gui et al., 2012; Huang, 2014; Song, 2020) comprising 910 patients, met the inclusion criteria (Figure 1 and Supplementary Material Characteristics of excluded studies). The included trials were published between 2001 and 2020, and all were from China.

### Description of Included Trials

All included trials studied patients with clinically diagnosed acute upper respiratory tract infection (AURTI—see Table 1). One was on the common cold in patients aged 16–37 years old (Huang, 2014), and the other six trials included children with fever without specifying the type of AURTI. One(Lv, 2010) study diagnosed AURTI according to the Chinese medicine clinical research guidelines (CMCRG) (China, M.o.H.o.t.p.s.R.o, 1997) and the diagnosis in Practical Pediatrics (Wu and Hu, 1995). Another study (Cao, 2012) diagnosed AURTI where the patient had “WBC<10.0×10^9^/L, neutrophils<0.70, fever, nasal congestion (or nasal discharge, cough, or dry mouth) red tongue, and yellow coating on the tongue, no complications, and onset within 24 h”. Others didn’t specify the diagnostic criteria. Only four studies (Cao, 2012; Gui et al., 2012; Huang, 2014; Song, 2020) specified a threshold of less than 24 h-5.5 days from the onset of symptoms for acute disease. Two trials (Cao, 2012; Gui et al., 2012) recruited patients from outpatient clinics, one from both outpatient clinics and inpatient wards (Lv, 2010), the remaining four did not specify the setting. Only two trials reported the source of funding (Lv, 2010; Huang, 2014), and both of them received funding from the government. None of the included trials stated whether or not a conflict of interest existed.

**TABLE 1:** Characteristics of included trials.

Study ID	Diagnosis (syndrome differentiation)	Setting	Funding sources	Course of symptoms: mean ± SD	Sample size (TG/CG)	Age: Mean ± SD (y)	Gender (Male/Total)	Name of the TG product and cointervention if available	Details of control group	Duration of treatment	Outcome measures
Cao (2012)	AURTI^a^	China, Clinic	NR	Within 24 h	109 (70/39)	NR, Children as reported	TG:34/70 CG:19/39	*Bupleuri radix* solution for injections (PO,1∼2 ml for children<3 years,2–3 ml for children≥3 years, tid)+ Usual care	Usual care: vitamin C (PO,0.1 g for children<3 years,0.2 g for children ≥3 years, tid), constant indoor temperature, maintains a level of humidity, physically cooling down when high fever occurs	72 h	systematic symptom resolution rate within 3 days from treatment onset^b^
Gui et al. (2012)	Infant, AURTI, fever	China, Clinic	NR	Within 24 h	108 (52/56)	42 days-12 m	TG:28/52 CG:30/56	*Bupleuri radix* solution for injections (aerosol inhalation for 15 min,2 ml, 1/d)+ Usual care	Usual care: Nebulised ribavirin (aerosol inhalation for 15 min, 10–15 mg/(kg.d), 1/d), penicillin (for children with increased leukocytes and neutrophils), metamizole sodium (if necessary)	5 d	3 days symptom resolution rate cough, nasal discharge),1d-2 days-3 days temperature, adverse effect
Huang (2014)	viral cold	China	Hainan Provincial Administration of Traditional Chinese Medicine	2–5.5 (2.46士0.37)d	80 (54/26)	16-37 (22.0士2.2)	NR	*Bupleuri radix* (raw herb) (15g, Decoction, 2/d, PO)	Ribavirin (30 mg,1/d)	7 d	Cure rate^c^
Lv (2010)	AURTI, fever	China, Clinic and ward	Taishan (Shandong province) Science and Technology Development Plan	NR	253 (100/50/52/51)	NR, Children as reported	NR	T1:*Bupleuri radix* injection (acupoint injection, 0.4 ml for children 1-2 years,0.6 ml for children 3-6 years, 1 ml for children 6 years,2/d) +Usual care T2:*Bupleuri radix* injection, intramuscular injection, regimens same as TG)+Usual care	C1:placebo (normal saline, acupoint injection, regimens same as TG)+Usual care, C2: Usual care: (ribavirin, intramuscular injection,10 mg/kg/d)+Usual care; Usual care: vitamin C、vitamin B6 (IV), metamizole sodium (intramuscular injection,10 mg/kg, if temperature >39 °C)	48 h (indicated by the outcome24–48 h symptom resolution)	Time to resolution (fever); 24–48 h symptom resolution rate (fever)
Song (2020)	Febrile children, caused by AURTI	China	NR	2h-3 d	100 (50/50)	5-14 y	TG:29/50 CG:27/50	*Bupleuri radix* (pill, 25mg/kg, PO)	Acetaminophen (PO,5–10 mg/kg, if temperature >38.5 C)	3 d	30min-60min–120min temperature, 3 days global symptom resolution rate (GPCR)
Xu and Mao (2001)	AURTIs, hyperpyrexia (rectal temperature:39.5–40.8°C)	China	NR	NR	140 (78/62)	NR, children as reported	TG:42/78 CG:38/62	*Bupleuri radix* injection, IM, 1 ml for children <3 years, 2 ml for children >3 years)+ Usual care	Usual care:acetaminophen (Suspension,10–15 mg/kg, PO)	1 d	1h-2h-4h–8 h temperature
Li et al. (2011)	Febrile children, caused by AURTI (temperature:38.5–40.0	China	NR	NR	120 (60/60)	1-8 y	TG:32/60 CG:34/60	*Bupleuri radix* (pills, 25mg/kg, PO, if temperature >38.5°C)+ Usual care	Acetaminophen (po,5–10 mg/kg, if temperature >38.5 °C) + Usual care Usual care: antibacterial drugs (in compliance with the “Guiding Principles for the Clinical Application of Antibacterial Drugs”), antiviral drugs etc.	96 h (indicated by the outcome 48–96 h symptom resolution)	30mim-1h–2 h temperature, 4 days global symptom resolution rate

a Diagnosed AURTI, with the criteria: WBC<10.0 × 109/L, neutrophils<0.70, fever, nasal congestion and discharge, cough, dry mouth, red tongue, yellow coating on the tongue, no complications, onset within 24 h.

b Definition of global symptom resolution is “no symptom and normal temperature”.

c Definition of cure rate is “no symptoms or sign of ARTI, normal laboratory checks”.

AURTI: acute upper respiratory tract infection; PO: oral; NR: not reported, TG: treatment group, CG: control group, SD: standard deviation, y: year, m: month, d: day, h: hour.

Comparisons were usual care or placebo. All trials involving usual care included some form of active intervention such as antibacterial drugs, antivirals, vitamins, or antipyretics.

Treatment duration in the included clinical trials ranged from 3 to 7 days.

One trial (Lv, 2010) was multi-armed comparing *Bupleuri radix* injection with acupoint injection and intramuscular injection versus placebo (acupoint injection with saline solution), and usual care. All others were two-arm parallel trials, of which one (Cao, 2012) used *Bupleuri radix* injection solution taken orally, one (Gui et al., 2012) used inhaled nebulized *Bupleuri radix* solution, one (Xu and Mao, 2001) used *Bupleuri radix* injection for intramuscular injection, two (Li et al., 2011; Song, 2020) used oral pills, one (Huang, 2014) used a decoction of *Bupleuri radix* pieces. Included trials seldom reported manufacturing or quality control details (see Table 2).

**TABLE 2:** Details of *Bupleuri radix* preparations in the included studies.

For patent proprietary CHM formulas						
Study ID	Preparations	Reference to publicly available materials, such as pharmacopeia, for the details about the composition, dosage, efficacy, safety	Details of the formula, namely 1) the proprietary product name (i.e., brand name), 2) name of manufacturer, 3) lot number, 4) production date and expiry date, 5) name and percentage of added materials	Statement of whether the patent proprietary formula used in the trial is for a condition that is identical to the publicly available reference(Y/N)	Chemical analysis reported? (Y/N)	Quality control reported? (Y/N)
Cao (2012)	Injection	Take l000 g of Bupleuri radix (raw herb), cut into sections, soak in water. After steam distillation, the initial distillate was collected, and then re-distillation was conducted to collect about 1,000 ml of the heavy distillate. Add 3 g polysorbide 80, stir to dissolve the oil completely, add 9 g sodium chloride, dissolve, filter, add water for injection with 1,000 ml, adjust pH value, then fine filtration, poach, and sterilize^a^	Prepared by Wanrong Sanjiu Pharmaceutical Co., LTD. (Shanxi, China) Added materials: 3 g polysorbide 80, 9 g sodium chloride, water Other information: NA	Y	N	N
Gui et al. (2012)	Injection		Added materials: 3 g polysorbide 80, 9 g sodium chloride, water Other information: NA	Y	N	N
Lv (2010)	Injection		Prepared by Shanxi Jinxin Shuanghe Pharmaceutical Co., LTD. (Shanxi, China) Added materials: 3 g polysorbide 80, 9 g sodium chloride, water Other information: NA	Y	N	N
Xu and Mao (2001)	Injection		Name and percentage of added materials: 3 g polysorbide 80, 9 g sodium chloride, water Other information: NA	Y	N	N
Song (2020), Li et al. (2011)	Pills	[Pharmacopeia] Bupleuri radix (raw herb) 3571 g, add water and decoct twice, filter the decoction, and concentrate the filtrate to a relative density of 1.15–1.20 C80°C), add ethanol to make the alcohol content reach 70%, add an appropriate amount of polyethylene glycol, heat to melt and mix. Makes 1000 g film-coated pill	Brand name: Bupleurum pill Prepared by Tianjin Tasly Pharmaceutical Co., LTD. (Tianjin, China) lot number:100206 SFDA approval number: Z19990,024 Name and percentage of added materials: water, ethanol, polyethylene glycol Other information: NA	Y	N	N

**For fixed CHM formulas**									
Study ID	Preparations	Name, source, processing method, and dosage of each medical substance	The authentication method of each ingredient and how, when, where, and by whom it was conducted; statement of whether any voucher specimen was retained, and if so, where they were kept and whether they are accessible	Principles, rationale, and interpretation of forming the formula	The production method of the formula	Safety assessment of the formula	References (s) as to the efficacy of the formula	Chemical analysis reported? (Y/N)	Quality control reported? (Y/N)
Huang (2014)	Decoction	Name: *Bupleuri radix* Other information: NA	*Bupleuri radix* (Chaihu) Other information: NA	Not relevant	Decoct with water	NA	NA	N	N

NA: Not available Y:Yes N:No SFDA: state food and drug administration.

a The *Bupleuri radix* injection used in the four studies may not be prepared by the same manufacturer. The process methods were extracted from Volume 17 Drug Standards for Traditional Chinese Medicine Patent Preparation issued by the Ministry of Health in 1998(NO. WS3-B-3297-98), which had been valid until 2011 (China, P.C.o.t.M.o.H.o.t.P.s.R.o, 1998).

The reported outcome measures included change in global symptoms (Li et al., 2011; Cao, 2012; Huang, 2014; Song, 2020) and relief of symptoms including nasal discharge (Gui et al., 2012), cough (Gui et al., 2012), and fever (Xu and Mao, 2001; Lv, 2010; Gui et al., 2012; Song, 2020). These were defined as time to complete resolution of the symptom, the severity of symptoms, or the number of patients resolved at a pre-defined time. One trial (Lv, 2010) reported the definition of global symptom resolution based on the “Guiding principles of clinical research on the treatment of children with exogenous fever by new Chinese medicine” (Medicine, 1994). Three trials that assessed global symptoms improvement, used self-defined criteria. Only two (Li et al., 2011; Gui et al., 2012) trials reported any information about adverse events. No trials reported antibiotic usage, length of hospitalization, and days off work or school. All the outcomes were measured during treatment or at the completion of treatment.

### Risk of Bias in Included Studies

**FIGURE 2:**
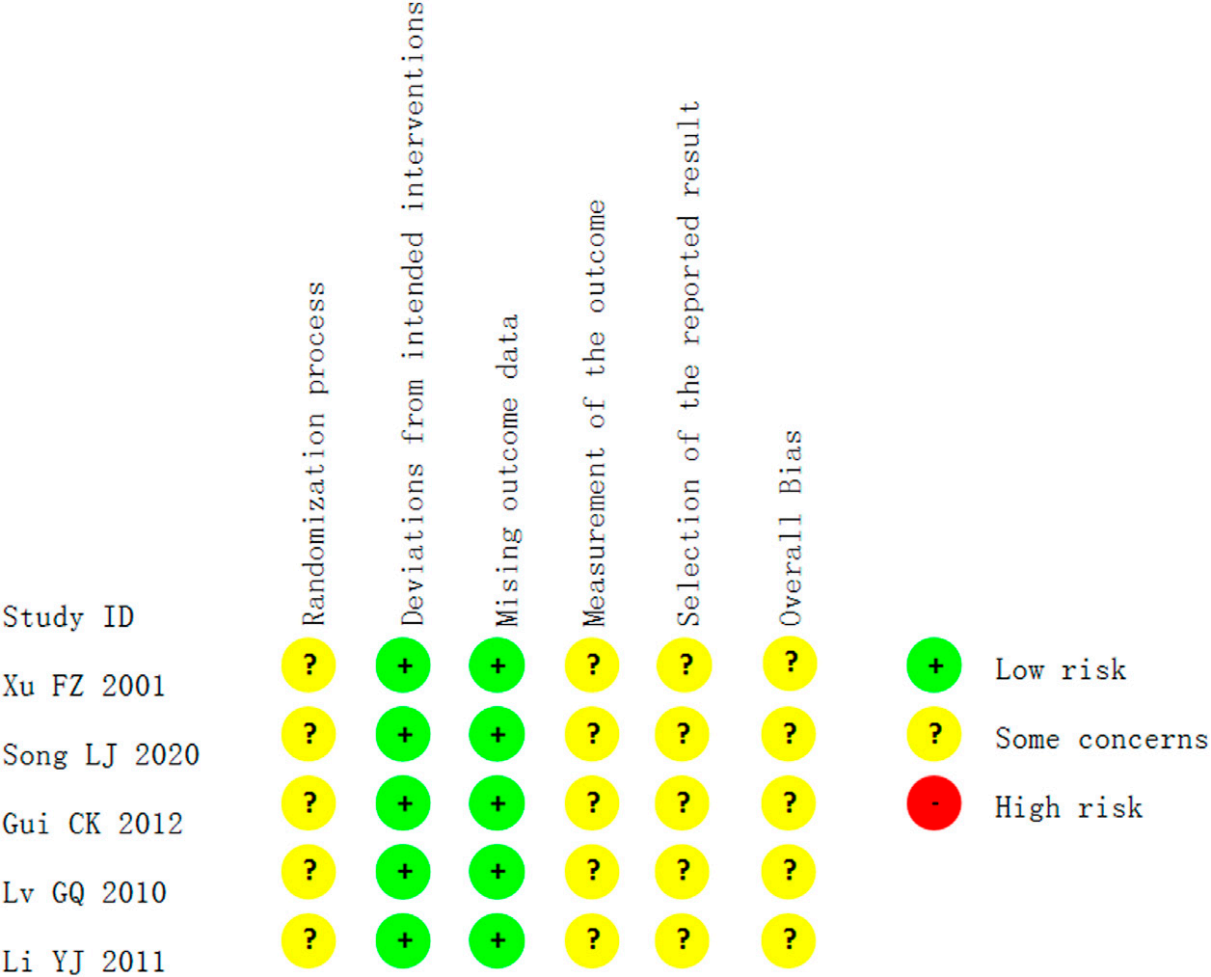
Risk of bias in the studies which reported the outcome of fever.

All the studies which reported the change in fever had “some concerns” in their risk of overall bias. All were described as “randomized,” one (Li et al., 2011) used the lottery method, randomly divided the participants into two groups according to odd and even numbers. Others did not report the method of random sequence generation or provided information on allocation concealment. Only one trial compared *Bupleuri radix* with placebo (Lv, 2010), there is a possibility that participants were aware of their assigned intervention during the trial, and there was no information about whether or not researchers were blinded. Researchers and participants in six trials (Xu and Mao, 2001; Lv, 2010; Li et al., 2011; Cao, 2012; Huang, 2014; Song, 2020) were likely aware of participants’ assigned intervention during the trial, as they assessed two interventions that were different in dosage, or form of preparation, or two types of interventions, or compared *Bupleuri radix* plus usual care versus usual care, without any blinding information given. No included trials reported any losses to follow-up; thus all were judged as low risk of bias in deviations from the intended intervention and missing outcome data. No trial provided sufficient information to determine whether blinding of outcome assessment was achieved. It was, therefore, assumed that assessors may be aware of the intervention received by study participants. Thus, there are “some concerns” regarding the measurement of the outcome. No trial had a protocol available, accordingly, there were “some concerns” of the risk of bias in the selection of the reported result (see Figure 2).

**FIGURE 3:**
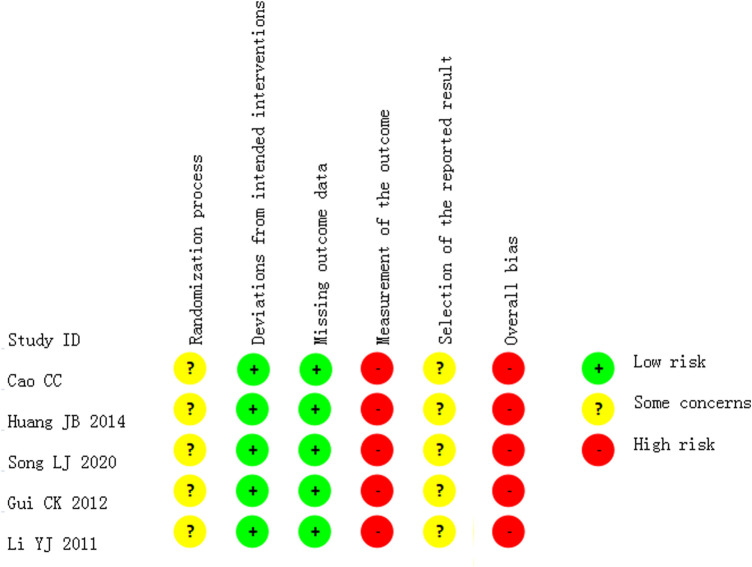
Risk of bias in the studies which reported the outcome of improvement in global symptoms or nasal discharge and cough.

All the studies which reported changes in global symptoms or nasal discharge and cough, had a high risk of overall bias, as all outcome measures were at risk of bias due to lack of blinding in the assessors (see Figure 3).

### Effects of Interventions

The included trials featured four comparison groups: *Bupleuri radix* plus Vitamins versus placebo plus Vitamins (Lv, 2010), *Bupleuri radix* plus usual care versus usual care (Xu and Mao, 2001; Cao, 2012; Gui et al., 2012), *Bupleuri radix* versus usual care (Huang, 2014; Song, 2020), *Bupleuri radix* plus usual care versus symptomatic treatment plus usual care (Lv, 2010; Li et al., 2011). Not all subgroup analyses were conducted due to insufficient data.

### *Bupleuri radix* Plus Usual Care Versus Placebo Plus Usual Care (1 Trial)

One trial (Lv, 2010), including 150 children with AURTI and fever, reported a statistically significant effect in favor of *Bupleuri radix* acupoint injection plus vitamins compared to placebo plus vitamins. The intervention shortened fever clearance time (FCT), defined as the time between the onset of treatment and sustained resolution of fever, i.e. the return of temperature to normal (<37.5°C) without recurrence during the same illness (MD: −33.32 h, 95%CI: −35.71, −30.93). It also improved the chance of fever resolution within 48 h (RR:14.00, 95%CI: 1.96, 99.94).

### *Bupleuri radix* Plus Usual Care Versus Usual Care (3 Trials)

One trial (Cao, 2012) including 109 children with AURTI showed no difference between oral administration of *Bupleuri radix* solution for injections plus vitamins, versus vitamins alone, in global symptom resolution rate within 3 days from treatment onset (RR:1.42, 95%CI: 0.73, 2.76). Another trial (Gui et al., 2012) including 108 infants with AURTI used nebulized inhalation of *Bupleuri radix* solution for injections plus nebulized ribavirin compared with nebulized ribavirin alone. It showed an equivalent effect in relieving the symptom of nasal discharge and cough. However, it showed significant treatment effects for temperature reduction. A third trial (Xu and Mao, 2001), including 140 children with ARTI and fever, assessed the change of temperature with time from treatment onset, and demonstrated intramuscular injection of *Bupleuri radix* injection plus oral acetaminophen when compared with oral acetaminophen alone had larger effects on temperature reduction. See Table 3.

**TABLE 3:** *Bupleuri radix* plus usual care versus usual care (3 trials).

Study ID	Participants	Intervention	Control	Outcomes	Measures	Effect estimate
Cao (2012)	109 AURTI children	*Bupleuri radix* solution for injections (po) plus vitamin C	Vitamin C	Global symptom resolution rate within 3 days from treatment onset	RR (95% CI)	1.42 [0.73, 2.76]
Gui et al. (2012)	108 AURTI infants	*Bupleuri radix* solution for injections (nebulized inhalation) plus nebulized ribavirin	Nebulised ribavirin	Resolution rate of nasal discharge within 3 days from treatment onset	RR (95% CI)	1.13 [0.70, 1.83]
				Resolution rate of cough within 3 days from treatment onset	RR (95% CI)	1.62 [0.80, 3.27]
				The temperature at day 1 from treatment onset	MD (95% CI)	−1.00°C [−1.19, −0.81]
				The temperature at day 2 from treatment onset	MD (95% CI)	−0.60°C [−0.77, −0.43]
				The temperature at day 3 from treatment onset	MD (95% CI)	−0.10°C [−0.23, 0.03]
Xu and Mao (2001)	140 children with AURTI and fever	*Bupleuri radix* injection (IM) plus oral acetaminophen	Oral acetaminophen	The temperature at 1st hour from treatment onset	MD (95% CI)	−0.27°C [−0.47, −0.07]
				The temperature at 2 nd h from treatment onset	MD (95% CI)	−0.41°C [−0.61, −0.21]
				The temperature at the 4th hour from treatment onset	MD (95% CI)	−0.10°C [−0.28, 0.08]
				The temperature at the 8th hour from treatment onset	MD (95% CI)	−0.74°C [−0.96, −0.52]

AURTI: acute upper respiratory tract infection; PO: oral; IM: intramuscular injection; RR: risk ratio; MD: mean difference; CI: confidence interval.

### *Bupleuri radix* Versus Usual Care (2 Trials)

One trial (Huang, 2014) including 80 adults with the common cold, showed the effect of *Bupleuri radix* decoction on cure rate (no symptom or sign of AURTI, normal laboratory checks) after 7 days of continuous treatment was no different to ribavirin. Another trial (Song, 2020) including 100 AURTI children with fever, showed that *Bupleuri radix* pills when compared with acetaminophen had an inferior effect on temperature reduction at 30 min from treatment onset, but there wasn’t a statistically significant effect at 60 min or 120 min from treatment onset. After 3 days of treatment, the pills demonstrated a higher global symptom resolution rate. See Table 4.

**TABLE 4:** *Bupleuri radix* versus usual care (2 trial).

Study ID	Participants	Intervention	Control	Outcomes	Measures	Effect estimate
Huang (2014)	80 adults with the common cold	*Bupleuri radix* decoction (po)	Ribavirin	Cure rate after 7 days continuous treatment	RR (95% CI)	0.78 [0.42, 1.43]
Song (2020)	100 children with AURTI and fever	*Bupleuri radix* pill (po)	Acetaminophen	Global symptom resolution rate within 3 days from treatment onset	RR (95% CI)	2.23 [1.32, 3.77]
				Temperature at 30 min from treatment onset	MD (95% CI)	0.13°C [0.03, 0.23]
				Temperature at 1st hour from treatment onset	MD (95% CI)	0.04°C [−0.05, 0.13]
				The temperature at 2 nd h from treatment onset	MD (95% CI)	0.07°C [−0.03, 0.17]

AURTI: acute upper respiratory tract infection; PO: oral; RR: risk ratio; MD: Mean Difference; CI: Confidence interval.

### *Bupleuri radix* Plus Usual Care Versus Symptomatic Treatment Plus Usual Care (2 Trials)

One trial (Lv, 2010) including 103 children with AURTI and fever reported no significant difference in Fever Clearance Time FCT (MD: 0.99h, 95%CI: −6.31, 4.33) and the chance of fever resolution within 48 h (RR: 0.78, 95%CI: 0.22, 2.76), when comparing *Bupleuri radix* injection (intramuscular injection) plus vitamins with ribavirin plus vitamins. The other trial (Li et al., 2011) including 120 children with AURTI and fever compared *Bupleuri radix* pills plus usual care (antibacterial and antiviral drugs) with acetaminophen plus usual care, also had a similar result on improving the symptom of fever, regarding the temperature at 30 min from treatment onset (MD: −0.03°C, 95%CI: −0.14, 0.08), the temperature at 1st hour (MD: 0.05°C, 95%CI: −0.12, 0.22), the temperature at 2nd h (MD: −0.04 °C, 95%CI: −0.16, 0.08). Moreover, *Bupleuri radix* pills plus usual care also failed to show a statistically significant effect on global symptom resolution rate within 4 days from treatment onset (RR: 1.25, 95%CI: 0.89, 1.76).

### Adverse Events

Only two trials reported the occurrence of AEs; one of which (Gui et al., 2012) reported that there were no AEs in either the intervention or control groups. The other one (Li et al., 2011) reported 2 cases had skin rash, 2 cases sweated profusely in the control group. The other trials did not report whether or not there were any AEs.

### Certainty Assessment of Evidence Using GRADE

All outcomes were evaluated as low-certainty or very low-certainty using the GRADE system approach. The details of the certainty of the available evidence can be found in Supplementary Tables. The certainty of the evidence was downgraded mainly due to the following reasons: 1) Risk of bias (high risk of detection bias and/or attrition bias); and 2) imprecision (small sample size or only one trial was included).

## Discussion

### Summary of Main Results

Seven randomized trials involving 910 patients with AURTI were included in this review, with no language restrictions. The review identified three *Bupleuri radix* preparations (*Bupleuri radix* solution for injections, pills, decoction) with four administration routes: oral, acupoint injection, intramuscular injection, and nebulized inhalation. There were no trials that evaluated the effectiveness and safety of *Bupleuri radix* injection, with the administration routes of oral, acupoint injection, intramuscular injection, and nebulized inhalation, according to the Ministry of Health 2011 standards. *Bupleuri radix* acupoint injection demonstrated a statistically significant effect in reducing fever when compared with placebo, measured by both fever clearance time (MD: −33.32 h, 95%CI: −35.71, −30.93), and the chance of fever resolution within a predefined time after treatment (RR:14, 95%CI:1.96, 99.94). Nebulised inhalation of *Bupleuri radix* solution for injections was more effective when combined with usual care in improving fever, compared with usual care alone. However, *Bupleuri radix* injection (intramuscular injection) plus vitamins failed to demonstrate an effect in reducing fever, when compared with ribavirin plus vitamins. *Bupleuri radix* pills showed similar antipyretic effects with acetaminophen (mean temperature at 30 min from treatment onset: 38.61 ± 0.26; at 1st hour: 37.51 ± 0.24; at 2nd hour: 36.74 ± 0.24) (Song, 2020). Included trials also suggested that oral administration of *Bupleuri radix* solution for injections, pills, and *Bupleuri radix* decoction have a similar effect on improving global AURTI symptoms and two key symptoms (nasal discharge and cough), when compared with usual care. Some usual care involved in the included trials were not usual care in the UK and many other countries, such as nebulized ribavirin is not usual care for the common cold. However, in China, they were usual care as some form of active intervention at the time of trials conducted. To facilitate the sorting of the results, they were all named “usual care”. Only two trials reported on the occurrence of adverse events; they did not record any adverse events in the intervention groups.

### Quality of the Evidence

There were no high-quality trials of *Bupleuri radix* for ARTI, and the quality of reporting was poor for all trials. None of the included trials were blinded; reporting indicated that the outcomes could have been influenced by a lack of blinding, and consequently were rated at a high risk of bias. There were no diagnostic or inclusion/exclusion criteria provided in most of the studies. This may have impacted the homogeneity of participants included in the trials, and the severity of symptoms. This makes the trials difficult to replicate.

As a natural product, the source of herb and the production method (plant extraction and whether the product is standardized) determine the proportion of constituents and resulting dose, thus influencing the effectiveness and safety of *Bupleuri radix*. This review identified three *Bupleuri radix* preparations, but most of them did not provide manufacturing details to ensure the quality and consistency of the products. The quality of evidence using GRADE was low for fever-related outcomes and very low for other symptom-related outcomes.

### Strengths and Limitations

This is the first systematic review evaluating the effects of *Bupleuri radix* on ARTI*.* The Cochrane methodology was followed and a protocol of this systematic review was registered and published online. We searched for RCTs using a broad search of different databases without language restrictions. Although we performed a broad search for ARTI in both children and adults, only trials involving AURTI qualified for inclusion. There were no trials on acute lower respiratory tract infections such as acute bronchitis. As all authors running the searches are Chinese, there may be a bias in that studies published in Chinese or Chinese journals, may have been more likely to be identified than articles in other non-English languages, even though language restrictions were not applied.

### Comparison With Other Studies or Reviews

Contemporary experimental research suggests that *Bupleuri radix* acts on various targets and pathways to produce significant antiviral effects on ARTI, but it is usually used with other herbals such as *Scutellariae Radix*, which are reported to be effective in improving the AURTI symptoms, such as fever and cough (Li and Deng, 2018; Wei et al., 2021). However, this review only included *Bupleuri radix* as a single herb and it failed to detect a statistically significant effect on global symptom resolution rate and some respiratory symptoms including cough and nasal discharge. Moreover, *Bupleuri radix* has been reported to exhibit mild to a severe adverse drug reaction or adverse events (ADR/AE) such as anaphylactic shock, acute hepatitis, and acute hepatic necrosis (Kong et al., 2010). However, the studies included in this review reported no side effects. Due to the relatively small number of studies combined with the short duration of treatment included in this review, we cannot draw any conclusions about the safety of *Bupleuri radix*.

### Implications for Future Studies

Future well-designed trials evaluating effectiveness and safety of *Bupleuri radix* for AURTI and reported according to the CONSORT checklist (Schulz et al., 2010) are vital. The potential for antibiotic sparing should also be studied in future trials. Concerning the assessment of safety, other data sources may be necessary to complement our findings.

### Implications for Practice

This review shows that *Bupleuri radix* may have a superior antipyretic effect on febrile adults or children who suffer from AURTI compared with placebo and usual care. However, fever management in AURTI with antipyretic drugs remains a common practice. There is currently insufficient evidence to recommend a change in practice.

## Conclusion

Low-certainty or very low-certainty evidence demonstrated that *Bupleuri radix* pill and solution for injections may have an antipyretic effect on febrile patients who suffer from AURTI, but it failed to show effects on other AURTI symptoms. However, the quality of included trials was generally low as many were poorly designed and inadequately blinded. Insufficient adverse event data was available to comment on its safety. Therefore, we could not draw more firm conclusions.

## Data Availability

The original contributions presented in the study are included in the article/Supplementary Material, further inquiries can be directed to the corresponding authors.
