# Supplementary material for: Bupleuri radix for Acute Uncomplicated Respiratory Tract Infection: A Systematic Review of Randomized Controlled Trials
Source: Front Pharmacol. 2022 Feb 4;12:787084. doi: 10.3389/fphar.2021.787084 (PMC8855037; doi:10.3389/fphar.2021.787084)
Supplement: Supplementary file 1 [file Table1.DOCX]

**China National Knowledge Infrastructure：**

(SU=('呼吸道感染'+'鼻炎'+'鼻窦炎'+'咽炎'+'喉炎'+'扁桃体炎'+'气管炎'+'卡他'+'咳'+'嗽'+'外感'+'感冒'+'流感'+'喉咙痛'+'咽痛'+'咽喉痛'+'咽痒'+'鼻塞'+'流涕'+'喘'+'肺'+'鼻'+'咽'+'喉') OR TKA = ('呼吸道感染'+'鼻炎'+'鼻窦炎'+'咽炎'+'喉炎'+'扁桃体炎'+'气管炎'+'卡他'+'咳'+'嗽'+'外感'+'感冒'+'流感'+'喉咙痛'+'咽痛'+'咽喉痛'+'咽痒'+'鼻塞'+'流涕'+'喘'+'肺'+'鼻'+'咽'+'喉')) AND (SU=('柴胡'+'茈胡'+'地薰'+'山菜'+'茹草'+'柴草'+'茨胡') OR TKA=('柴胡'+'茈胡'+'地薰'+'山菜'+'茹草'+'柴草'+'茨胡') ) AND FT = '对照' NOT TI = ('小柴胡' + '正柴胡'+ '大柴胡'+ '柴胡桂枝汤'+'柴胡达原饮'+'柴胡疏肝散') 1784

**SU=主题词**

**WanFang:**

主题:('呼吸道感染'or '鼻炎'or '鼻窦炎' or '咽炎' or '喉炎' or '扁桃体炎' or '气管炎' or '卡他' or '咳' or '嗽' or '外感' or '感冒' or '流感' or '喉咙痛' or '咽痛' or '咽喉痛' or '咽痒' or '鼻塞' or '流涕' or '喘' or '肺' or '鼻' or '咽' or '喉') and 主题:("柴胡"or '茈胡' or '地薰' or '山菜' or '茹草' or '柴草' or "茨胡") not 题名:('小柴胡' or '大柴胡' or '柴胡桂枝汤'or '柴胡达原饮' or '柴胡疏肝散' or '正柴胡') 181

**VIP:**

R=('呼吸道感染'+'鼻炎'+'鼻窦炎'+'咽炎'+'喉炎'+'扁桃体炎'+'气管炎'+'卡他'+'咳'+'嗽'+'外感'+'感冒'+'流感'+'喉咙痛'+'咽痛'+'咽喉痛'+'咽痒'+'鼻塞'+'流涕'+'喘'+'肺'+'鼻'+'咽'+'喉') AND R=(柴胡+茈胡+ 地薰+山菜+茹草+柴草+茨胡) NOT M=('小柴胡' or '大柴胡' or '柴胡桂枝汤'or '柴胡达原饮' or '柴胡疏肝散' or '正柴胡)

R=文摘

U=任意字段 1549

**CBM：**

| 9 | ((#8) NOT (#7)) |
| --- | --- |
| 8 | (#6) AND (#4) |
| 7 | "小柴胡"[标题:智能] OR "大柴胡"[标题:智能] OR "柴胡桂枝汤"[标题:智能] OR "柴胡达原饮"[标题:智能] OR "柴胡疏肝散"[标题:智能] OR "正柴胡"[标题:智能] |
| 6 | (#5) OR (#1) |
| 5 | "柴胡"[常用字段:智能] OR "茈胡"[常用字段:智能] OR "地薰"[常用字段:智能] OR "山菜"[常用字段:智能] OR "茹草"[常用字段:智能] OR "柴草"[常用字段:智能] OR "茨胡"[常用字段:智能] |
| 4 | (#3) OR (#2) |
| 3 | 1）"呼吸道感染"[常用字段:智能] OR "感冒"[常用字段:智能] OR "流感"[常用字段:智能] OR "嗽"[常用字段:智能] OR "鼻炎"[常用字段:智能] OR "鼻窦炎"[常用字段:智能] OR "咳"[常用字段:智能] OR"外感"[常用字段:智能] OR"喉咙痛"[常用字段:智能] OR "咽痛"[常用字段:智能] OR "咽喉痛"[常用字段:智能] OR "咽痒"[常用字段:智能] OR "咽炎"[常用字段:智能] OR "喉炎"[常用字段:智能] OR "鼻塞"[常用字段:智能] OR "流涕"[常用字段:智能] OR "卡他"[常用字段:智能] OR "支气管炎"[常用字段:智能] OR "扁桃体炎"[常用字段:智能] OR "流感"[常用字段:智能] OR "喘"[常用字段:智能] OR "肺"[常用字段:智能] OR "鼻"[常用字段:智能] OR "咽"[常用字段:智能] OR "喉"[常用字段:智能] |
| 2 | 呼吸道感染 |
| 1 | "柴胡"[不加权:扩展] |

**EMBASE:**

#1 'respiratory tract infection'/exp

#2 'respiratory tract inflammation'/exp

#3 'common cold'/exp

#4 'supraglottitis'/exp

#5 'laryngitis'/exp

#6 'influenza, human'/exp

#7 'tracheitis'/exp

#8 'bronchitis'/exp

#9 'pharyngitis'/exp

#10 'rhinitis'/exp

#11 'sinusitis'/exp

#12 (respiratory tract infection* or RTI* or (chest NEAR/3 infection*) or rhinit* or sinusit* or pharyngit* or laryngit* or rhinosinusit* or rhinopharyngit* or rhinolaryngit* or nasosinusit* or nasopharyngit* or nasolaryngit* or sinonasal* or rhino-sinusit* or rhino-pharyngit* or rhino-laryngit* or naso-sinusit* or naso-pharyngit* or sino-nasal* or cold* or bronchit* or supraglottit* or epiglott* or pulmon* or tracheit* or respirat* or tonsillit* or flu or influenza or cough* or sneez*or catarrh or muc?us or phlegm* or (sore NEAR/3 throat) or (throat NEAR/3 pain) or (blocked NEAR/3 nose) or (runn* NEAR/3 nose) or (stuff* NEAR/3 nose) or (short* NEAR/3 breath*) or breathless* or rhinorrh?ea or congest* or discharge* or (tight NEAR/3 chest)).mp.

#12 #1 or #2 or #3 or #4 or #5 or #6 or #7 or #8 or #9 or #10 or #11 or #12

#13 'bupleurum'/exp

#14 hare*:ab,ti AND ear*:ab,ti

#15 bupleurum:ab,ti OR thorowax:ab,ti OR saiko*:ab,ti OR chai:ab,ti OR saikosaponin:ab,ti OR chaihu:ab,ti OR bupleuri:ab,ti OR triterpenoid:ab,ti OR saponins:ab,ti

#16 #13 or #14 or #15

#17 random

#18 #12 and #16 and #17

[mp=title, abstract, original title, name of substance word, subject heading word, keyword

heading word, protocol supplementary concept word, rare disease supplementary concept

word, unique identifier, synonyms]

Pubmed:

1. Supraglottitis[MeSH Terms]

2. Laryngitis[MeSH Terms]

3. Cough[MeSH Terms]

4. Influenza, Human[MeSH Terms]

5. Tracheitis[MeSH Terms]

6. Bronchitis[MeSH Terms]

7. Pharyngitis[MeSH Terms]

8. Rhinitis[MeSH Terms]

9. Sinusitis[MeSH Terms]

10. Common Cold[MeSH Terms]

11. Respiratory Tract Infections[MeSH Terms]

12. ((((((((((((((((((((((((respiratory tract infection*[Title/Abstract]) OR (respiratory adj3 infection*[Title/Abstract])) OR (RTI*[Title/Abstract])) OR (chest adj3 infection*[Title/Abstract])) OR (rhinit*[Title/Abstract])) OR (sinusit*[Title/Abstract])) OR (pharyngit*[Title/Abstract])) OR (laryngit*[Title/Abstract])) OR (rhinosinusit*[Title/Abstract])) OR (rhinopharyngit*[Title/Abstract])) OR (nasosinusit*[Title/Abstract])) OR (nasopharyngit*[Title/Abstract])) OR ( sinonasal*[Title/Abstract] OR rhino-sinusit*[Title/Abstract] OR rhino-pharyngit*[Title/Abstract] OR naso-sinusit*[Title/Abstract] OR naso-pharyngit*[Title/Abstract] OR sino-nasal*[Title/Abstract] OR cold*[Title/Abstract] OR bronchit*[Title/Abstract] OR supraglottit*[Title/Abstract] OR epiglott*[Title/Abstract] OR pulmon*[Title/Abstract] OR tracheit*[Title/Abstract] OR respirat*[Title/Abstract] OR tonsillit*[Title/Abstract] OR flu[Title/Abstract] OR influenza[Title/Abstract] OR cough*[Title/Abstract] OR catarrh[Title/Abstract] OR muc?us[Title/Abstract] OR phlegm*[Title/Abstract])) OR (sore adj3 throat[Title/Abstract])) OR (throat adj3 pain[Title/Abstract])) OR (blocked adj3 nose[Title/Abstract])) OR (runn* adj3 nose[Title/Abstract])) OR (stuff* adj3 nose[Title/Abstract])) OR (short* adj3 breath*[Title/Abstract])) OR (breathless*[Title/Abstract])) OR (rhinorrh?ea[Title/Abstract])) OR (congest*[Title/Abstract])) OR (discharge*[Title/Abstract])) OR (tight adj3 chest[Title/Abstract])) OR (sneez*[Title/Abstract])

13. 1 OR 2 OR 3 OR 4 OR 5 OR 6 OR 7 OR 8 OR 9 OR 10 OR 11 OR 12

14. ((((((((saiko*[Title/Abstract]) OR (chai[Title/Abstract])) OR (saikosaponin[Title/Abstract])) OR (bupleuri[Title/Abstract])) OR (triterpenoid[Title/Abstract])) OR (saponins[Title/Abstract])) OR ((hare*[Title/Abstract]) AND (ear*[Title/Abstract]))) OR ((bupleurum[Title/Abstract]) OR (thorowax[Title/Abstract]))) OR (bupleurum[MeSH Terms])

15. (((((saiko*[Title/Abstract]) OR (chai[Title/Abstract])) OR (saikosaponin[Title/Abstract])) OR (bupleuri[Title/Abstract])) OR (triterpenoid[Title/Abstract])) OR (saponins[Title/Abstract])

16. (hare*[Title/Abstract]) AND (ear*[Title/Abstract])

17. (bupleurum[Title/Abstract]) OR (thorowax[Title/Abstract])

18. bupleurum[MeSH Terms]

19. 14 OR 15 OR 16 OR 17 OR 18

20. Random

21. 13 AND 19 AND 20

CENTRAL

#1 (rhinit* or sinusit* or pharyngit* or laryngit* or rhinosinusit* or rhinopharyngit* or rhinolaryngit* or nasosinusit* or nasopharyngit* or nasolaryngit* or sinonasal* or rhinosinusit* or rhino-pharyngit* or rhino-laryngit* or naso-sinusit* or naso-pharyngit* or sino-nasal* or cold* or tonsillit*):ti,ab,kw (Word variations have been searched)

#2 (bronchit* or supraglottit* or epiglott* or pulmon* or tracheit* or respirat* or flu or influenza):ti,ab,kw (Word variations have been searched)

#3 ((cough* or sneez* or catarrh or muc?us or phlegm* or (sore near throat) or (throat near pain) or (blocked near nose) or (runn* near nose) or (stuff* near nose) or (short* near breath*) or breathless* or rhinorrh?ea or congest* or discharge* or (tight near chest))):ti,ab,kw (Word variations have been searched)

#4 (respiratory tract infection):ti,ab,kw (Word variations have been searched)

#5 MeSH descriptor: [Respiratory Tract Infections] explode all trees

#6 MeSH descriptor: [Laryngitis] explode all trees

#7 MeSH descriptor: [Bronchitis] explode all trees

#8 MeSH descriptor: [Rhinitis] explode all trees

#9 MeSH descriptor: [Rhinitis] explode all trees

#10 #1 or #2 or #3 or #4 or #5 or #6 or #7 or #8 or #9

#11 MeSH descriptor: [Bupleurum] explode all trees

#12 (hare* AND ear*):ti,ab,kw (Word variations have been searched)

#13 bupleurum:ab,ti,kw OR thorowax:ab,ti,kw OR saiko*:ab,ti,kw OR chai:ab,ti,kw OR saikosaponin:ab,ti,kw OR chaihu:ab,ti,kw OR bupleuri:ab,ti,kw OR triterpenoid:ab,ti,kw OR saponins:ab,ti,kw

#14 #11 or #12 or #13

#15 #10 and #14
