# Supplementary material for: Bupleuri radix for Acute Uncomplicated Respiratory Tract Infection: A Systematic Review of Randomized Controlled Trials
Source: Front Pharmacol. 2022 Feb 4;12:787084. doi: 10.3389/fphar.2021.787084 (PMC8855037; doi:10.3389/fphar.2021.787084)
Supplement: Supplementary file 2 [file Table3.DOC]

**Supplementary Table 1** **GRADE evaluation form of evidence certainty for *Bupleuri radix* plus usual care versus placebo plus usual care**

**Patient or population:** Patients with clinically diagnosed acute upper respiratory tract infection (AURTI)

**Comparison:** *Bupleuri radix* plus usual care versus placebo plus usual care

**Setting**: Clinc and ward

| **Certainty assessment** | | | | | | | **№ of patients** | | **Effect** | | **Certainty** |
| --- | --- | --- | --- | --- | --- | --- | --- | --- | --- | --- | --- |
| **№ of studies** | **Study design** | **Risk of bias** | **Inconsistency** | **Indirectness** | **Imprecision** | **Other considerations** | ***Bupleuri radix* plus usual care** | **Placebo plus usual care** | **Relative (95% CI)** | **Absolute (95% CI)** |
| **The chance of fever resolution within 48h** | | | | | | | | | | | |
| 1 | randomised trials | serious ^a,b^ | not serious | not serious | serious ^c^ | none | 28/100 (28.0%) | 1/50 (2.0%) | **RR 14.00** (1.96 to 99.94) | **260 more per 1,000** (from 19 more to 1,000 more) | ⨁⨁◯◯ LOW |
| **Fever clearance time (FCT)** | | | | | | | | | | | |
| 1 | randomised trials | serious ^a,b^ | not serious | not serious | serious ^c^ | none | 100 | 50 | - | MD **33.32 lower** (35.71 lower to 30.93 lower) | ⨁⨁◯◯ LOW |

**CI:** Confidence interval; **RR:** Risk ratio; **MD:** Mean difference

#### Explanations

a. Some concerns of the risk of bias in selection of the reported result

b. Did not report the method of random sequence generation, provided information on allocation concealment

c. Small sample size and only one trial were included

**Supplementary Table 2** **GRADE evaluation form of evidence certainty for *Bupleuri radix* plus usual care versus usual care**

**Patient or population:** Patients with clinically diagnosed acute upper respiratory tract infection (AURTI)

**Comparison:** *Bupleuri radix* plus usual care versus usual care

**Setting**: Clinc

| **Certainty assessment** | | | | | | | **№ of patients** | | **Effect** | | **Certainty** |
| --- | --- | --- | --- | --- | --- | --- | --- | --- | --- | --- | --- |
| **№ of studies** | **Study design** | **Risk of bias** | **Inconsistency** | **Indirectness** | **Imprecision** | **Other considerations** | ***Bupleuri radix* plus usual care** | **Usual care** | **Relative (95% CI)** | **Absolute (95% CI)** |
| **Global symptom resolution rate within 3 days from treatment onset** | | | | | | | | | | | |
| 1 | Randomised trials | very serious ^a,b^ | not serious | not serious | serious ^c^ | none | 23/70 (32.9%) | 9/39 (23.1%) | **RR 1.42** (0.73 to 2.76) | **97 more per 1,000** (from 62 fewer to 406 more) | ⨁◯◯◯ VERY LOW |
| **Resolution rate of nasal discharge within 3 days from treatment onset** | | | | | | | | | | | |
| 1 | Randomised trials | very serious ^b^ | not serious | not serious | serious ^c^ | none | 21/52 (40.4%) | 20/56 (35.7%) | **RR 1.13** (0.70 to 1.83) | **46 more per 1,000** (from 107 fewer to 296 more) | ⨁◯◯◯ VERY LOW |
| **Resolution rate of cough within 3 days from treatment onset** | | | | | | | | | | | |
| 1 | Randomised trials | very serious ^b^ | not serious | not serious | serious ^c^ | none | 15/52 (28.8%) | 10/56 (17.9%) | **RR 1.62** (0.80 to 3.27) | **111 more per 1,000** (from 36 fewer to 405 more) | ⨁◯◯◯ VERY LOW |
| **Temperature at day 1 from treatment onset** | | | | | | | | | | | |
| 1 | Randomised trials | serious ^d,e^ | not serious | not serious | serious ^c^ | none | 52 | 56 | - | MD **1.0 lower** (1.19 lower to 0.81 lower) | ⨁⨁◯◯ LOW |
| **Temperature at day 2 from treatment onset** | | | | | | | | | | | |
| 1 | randomised trials | serious ^d,e^ | not serious | not serious | serious ^c^ | none | 52 | 56 | - | MD **0.6 lower** (0.77 lower to 0.43 lower) | ⨁⨁◯◯ LOW |
| **Temperature at day 3 from treatment onset** | | | | | | | | | | | |
| 1 | randomised trials | serious ^d,e^ | not serious | not serious | serious ^c^ | none | 52 | 56 | - | MD **0.1 lower** (0.23 lower to 0.03 higher) | ⨁⨁◯◯ LOW |
| **Temperature at 1st hour from treatment onset** | | | | | | | | | | | |
| 1 | randomised trials | serious ^d,e^ | not serious | not serious | serious ^c^ | none | 78 | 62 | - | MD **0.27 lower** (0.47 lower to 0.07 lower) | ⨁⨁◯◯ LOW |
| **Temperature at 2nd hour from treatment onset** | | | | | | | | | | | |
| 1 | randomised trials | serious ^d,e^ | not serious | not serious | serious ^c^ | none | 78 | 62 | - | MD **0.41 lower** (0.61 lower to 0.21 lower) | ⨁⨁◯◯ LOW |
| **Temperature at 4th hour from treatment onset** | | | | | | | | | | | |
| 1 | randomised trials | serious ^d,e^ | not serious | not serious | serious ^c^ | none | 78 | 62 | - | MD **0.1 lower** (0.28 lower to 0.08 higher) | ⨁⨁◯◯ LOW |
| **Temperature at 8th hour from treatment onset** | | | | | | | | | | | |
| 1 | randomised trials | serious ^d,e^ | not serious | not serious | serious ^c^ | none | 78 | 87 | - | MD **0.74 lower** (0.96 lower to 0.52 lower) | ⨁⨁◯◯ LOW |

**CI:** Confidence interval; **RR:** Risk ratio; **MD:** Mean difference

#### Explanations

a. High risk in randomization process

b. High risk in measurement of the outcome

c. Small sample size and only one trial were included

d. Some concerns of the risk of bias in selection of the reported result

e. Did not report the method of random sequence generation, provided information on allocation concealment

**Supplementary Table 3** **GRADE evaluation form of evidence certainty for *Bupleuri radix* versus usual care**

**Patient or population:** Patients with clinically diagnosed acute upper respiratory tract infection (AURTI)

**Comparison:** *Bupleuri radix* versus usual care

**Setting**: Not provided

| **Certainty assessment** | | | | | | | **№ of patients** | | **Effect** | | **Certainty** |
| --- | --- | --- | --- | --- | --- | --- | --- | --- | --- | --- | --- |
| **№ of studies** | **Study design** | **Risk of bias** | **Inconsistency** | **Indirectness** | **Imprecision** | **Other considerations** | ***Bupleuri radix*** | **Usual care** | **Relative (95% CI)** | **Absolute (95% CI)** |
| **Cure rate after 7 days continuous treatment** | | | | | | | | | | | |
| 1 | randomised trials | very serious ^a,b^ | not serious | not serious | serious ^c^ | none | 9/26 (34.6%) | 24/54 (44.4%) | **RR 0.78** (0.42 to 1.43) | **98 fewer per 1,000** (from 258 fewer to 191 more) | ⨁◯◯◯ VERY LOW |
| **Systematic symptom resolution rate within 3 days from treatment onset** | | | | | | | | | | | |
| 1 | randomised trials | very serious ^a,b^ | not serious | not serious | serious ^c^ | none | 29/50 (58.0%) | 13/50 (26.0%) | **RR 2.23** (1.32 to 3.77) | **320 more per 1,000** (from 83 more to 720 more) | ⨁◯◯◯ VERY LOW |
| **Temperature at 30 min from treatment onset** | | | | | | | | | | | |
| 1 | randomised trials | serious ^b^ | not serious | not serious | serious ^c^ | none | 50 | 50 | - | MD **0.13 higher** (0.03 higher to 0.23 higher) | ⨁⨁◯◯ LOW |
| **Temperature at 1st hour from treatment onse** | | | | | | | | | | | |
| 1 | randomised trials | serious ^d,e^ | not serious | not serious | serious ^c^ | none | 50 | 50 | - | MD **0.04 higher** (0.05 lower to 0.13 higher) | ⨁⨁◯◯ LOW |
| **Temperature at 2nd hour from treatment onset** | | | | | | | | | | | |
| 1 | randomised trials | serious ^d,e^ | not serious | not serious | serious ^c^ | none | 50 | 50 | - | MD **0.07 higher** (0.03 lower to 0.17 higher) | ⨁⨁◯◯ LOW |

**CI:** Confidence interval; **RR:** Risk ratio; **MD:** Mean difference

#### Explanations

a. High risk in randomization process

b. High risk in measurement of the outcome

c. Small sample size and only one trial were included

d. Did not report the method of random sequence generation, provided information on allocation concealment

e. Some concerns of the risk of bias in selection of the reported result

**Supplementary Table 4** **GRADE evaluation form of evidence certainty for *Bupleuri radix* plus usual care versus symptomatic treatment plus usual care**

**Patient or population:** Patients with clinically diagnosed acute upper respiratory tract infection (AURTI)

**Comparison:** Bupleuri radix plus usual care versus ribavirin plus usual care

**Setting**: Clinc and ward

| **Certainty assessment** | | | | | | | **№ of patients** | | | **Effect** | | | **Certainty** | |
| --- | --- | --- | --- | --- | --- | --- | --- | --- | --- | --- | --- | --- | --- | --- |
| **№ of studies** | **Study design** | **Risk of bias** | **Inconsistency** | **Indirectness** | **Imprecision** | **Other considerations** | ***Bupleuri radix* plus usual care** | **Ribavirin plus usual care** | | **Relative (95% CI)** | | **Absolute (95% CI)** |
| **The chance of fever resolution within 48h** | | | | | | | | | | | | | | |
| 1 | randomised trials | serious ^a,b^ | not serious | not serious | serious ^c^ | none | 4/52 (7.7%) | 5/51 (9.8%) | | **RR 0.78** (0.22 to 2.76) | | **22 fewer per 1,000** (from 76 fewer to 173 more) | ⨁⨁◯◯ LOW | |
| **Fever clearance time (FCT)** | | | | | | | | | | | | | | |
| 1 | randomised trials | serious ^a,b^ | not serious | not serious | serious ^c^ | none | 52 | 51 | | - | | MD **0.99 lower** (6.31 lower to 4.33 higher) | ⨁⨁◯◯ LOW | |
| **Temperature at 30 min from treatment onset** | | | | | | | | | | | | | | |
| 1 | randomised trials | serious ^b^ | not serious | not serious | serious ^c^ | none | 60 | 60 | | | - | MD 0.03 lower  (0.14 lower to 0.08 higher) | | ⨁⨁◯◯ LOW |
| **Temperature at 1st hour from treatment onse** | | | | | | | | | | | | | | |
| 1 | randomised trials | serious ^d,e^ | not serious | not serious | serious ^c^ | none | 60 | 60 | | | - | MD 0.05 higher  (0.12 lower to 0.22 higher) | | ⨁⨁◯◯ LOW |
| **Temperature at 2nd hour from treatment onset** | | | | | | | | | | | | | | |
| 1 | randomised trials | serious ^d,e^ | not serious | not serious | serious ^c^ | none | 60 | 60 | | | - | MD 0.04 lower  (0.16 lower to 0.08 higher) | | ⨁⨁◯◯ LOW |
| **Global symptom resolution rate within 3 days from treatment onset** | | | | | | | | | | | | | | |
| 1 | Randomised trials | very serious ^a,b^ | not serious | not serious | serious ^c^ | none | 35/60 (58.3%) | 28/60 (46.7%) | RR 1.25  (0.89 to 1.76) | | | 58 more per 1,000  (from 25 fewer to 175 more) | | ⨁◯◯◯ VERY LOW |

**CI:** Confidence interval; **RR:** Risk ratio; **MD:** Mean difference

#### Explanations

a. Some concerns of the risk of bias in selection of the reported result

b. Did not report the method of random sequence generation, provided information on allocation concealment

c. Small sample size and only one trial were included
