# Supplementary material for: Bupleuri radix for Acute Uncomplicated Respiratory Tract Infection: A Systematic Review of Randomized Controlled Trials
Source: Front Pharmacol. 2022 Feb 4;12:787084. doi: 10.3389/fphar.2021.787084 (PMC8855037; doi:10.3389/fphar.2021.787084)
Supplement: Supplementary file 3 [file Table2.doc]

**Characteristics of excluded studies**

| **Study** | **Reason for exclusion** |
| --- | --- |
| Chen 2014^[1]^ | Not RCT |
| Zhao 2017^[2]^ | *Bupleuri radix* combined with other TCM therapies |
| Xu 2014^[3]^ | Not RCT |
| Chen 2010^[4]^ | Same as Lv GQ 2010 data, but Lv GQ 2010 data is more abundant |
| Zuo 2003^[5]^ | *Bupleuri radix* combined with other TCM therapies |
| Xu 2002^[6]^ | *Bupleuri radix* combined with other TCM therapies |
| Ni 2002^[7]^ | Not ARTI |
| Zheng 1995^[8]^ | Not RCT |

**References to studies excluded from this review**

[1] Cheng Xuehua. Observation on the curative effect of adding Chaihu decoction to the rectum to treat children with exogenous fever. Nursing Research, 2014,28(4):485-486. DOI:10.3969/j.issn.1009-6493.2014 .04.044.

[2] Zhao Wangsen, Zhang Yecong, Wu Mengdie, et al. Analysis of the clinical efficacy of Chaihu injection combined with oseltamivir in the treatment of flu-like symptoms. Systems Medicine, 2017, 2(20): 34-36. DOI: 10.19368/j.cnki.2096-1782.2017.20.034.

[3] Xu Qian, Li Jinghong. Observation on the curative effect of Chaihu sticking application on children with exopathic fever[J]. Gansu Medicine, 2014, 33(3): 191-193.

[4] Chen Qin. Observation and nursing care of the curative effect of Chaihu injection at Quchi acupoint in the treatment of children with exogenous fever. Inner Mongolia Traditional Chinese Medicine, 2010,29(15):177-177. DOI:10.3969/j.issn.1006 -0979.2010.15.164.

[5] Zuo Wenge, Ke Haihong. Chaihu dripping pills in the treatment of 103 cases of viral upper respiratory tract infection. Journal of Traditional Chinese Medicine, 2003, 44(4): 285. DOI: 10.3321/j.issn: 1001-1668.2003.04.029.

[6] Xu Jinsong, He Mingfeng, Liang Zhangrong. Clinical observation of Bupleurum and Houttuynia injection combined with penicillin in the treatment of exopathic hyperthermia. Journal of Guiyang College of Traditional Chinese Medicine, 2002, 24(4): 18-19. DOI :10.3969/j.issn.1002-1108.2002.04.013.

[7] Ni Meiyuan, Gao Meixia. Development of Chaihu nasal drops and observation of clinical efficacy. Shandong Pharmaceutical Industry, 2002, 21(4): 37-38. DOI:10.3969/j.issn.1672-7738.2002. 04.037.

[8] Zheng Xiurong. Observation of 145 cases of secondary Bupleurum and thymosensitivity in the treatment of upper sensation. Journal of Mathematical Medicine, 1995, (01), 37-38
